# Evaluation of yield attributes and bioactive phytochemicals of twenty amaranth genotypes of Bengal floodplain

**DOI:** 10.1016/j.heliyon.2023.e19644

**Published:** 2023-08-30

**Authors:** Nishat Jahan, Umakanta Sarker, Mohammad Mehfuz Hasan Saikat, Md Motaher Hossain, Mohammad Golam Azam, Daoud Ali, Sezai Ercisli, Kirill S. Golokhvast

**Affiliations:** a Department of Genetics and Plant Breeding, Faculty of Agriculture, Bangabandhu Sheikh Mujibur Rahman Agricultural University, Gazipur, 1706, Bangladesh; b Department of Plant Pathology, Faculty of Agriculture, Bangabandhu Sheikh Mujibur Rahman Agricultural University, Gazipur, 1706, Bangladesh; c Pulses Research Centre, Bangladesh Agricultural Research Institute, Bangladesh, 6620; d Department of Genetics and Plant Breeding, Faculty of Agriculture, Bangladesh Agricultural University, Mymensingh, Bangladesh, 2202; e Department of Zoology, College of Science, King Saud University, P.O. Box 2455, Riyadh, 11451, Saudi Arabia; f Department of Horticulture, Faculty of Agriculture, Ataturk University, 25240, Erzurum, Turkey; g HGF Agro, Ata Teknokent, TR-25240, Erzurum, Turkey; h Siberian Federal Scientific Center of Agrobiotechnology RAS, 2b Centralnaya, Krasnoobsk, 630501, Russia

**Keywords:** Colorant pigments, Correlation, Dendrogram, Foliage yield, Heatmap, Heritability, Path analysis, Principal component analysis, Total polyphenols and flavonoids, Variability

## Abstract

Twenty vegetable amaranth (VA) genotypes were evaluated to assess the variability, associations, path coefficient, and principal component analysis (PCA) of morpho-chemical traits. The genotypes exhibited adequate antioxidant colorants, phytochemicals, and antiradical capacity with significant variations across genotypes. Genetic parameters revealed selection criteria for the majority of the traits for improving amaranth foliage yield (FY). Based on correlation coefficient, stem weight, stem base diameter, root weight, plant height, and shoot weight for significant development of FY of VA. Observing significant genotypic correlation with high positive direct effects on FY, the path coefficient (PC) of root weight, stem base diameter, stem weight, and shoot weight could contribute to the noteworthy development of FY of VA. The genotypes AA5, AA6, AA8, AA10, AA11, AA19, and AA20 might be selected for high FY, antioxidant colorants, and antiradical phytochemicals to utilize as colorants and antiradical rich superior high yielding cultivars. The first PC accounted for 37.8% of the variances, which implies a larger proportion of variable information explained by PC1. The features that contributed more to PC1 were FY, SW, STW, RW, and PH, whereas Chl *b*, total Chl, and Chl *a* contributed to the second PC. This suggests that there are significant genetic differences between amaranths in terms of biochemical and agro-morphological characteristics. The findings of the current work support plant breeders to investigate the genetic potential of the amaranth germplasm, notably in biochemical parameters. High colorants enrich genotypes that can be selected for extracting natural colorants to use in food processing industries.

## 1 Introduction

Vegetables in particular are extremely beneficial to human health since they are perfect foods with a wide array of nutrients. Amaranth compared to other vegetables, is a low-priced source because of its high yield and low production cost. Sometimes, it is considered a poor's man vegetable. It contains high-quality and quantity protein with essential amino acids, like lysine and methionine, carotenoids, and dietary fiber [1,2]. Vegetables' leaves are enriched in vitamins, and pigments [[3], [4], [5]], in addition to minerals like calcium, magnesium, potassium, phosphorus, iron, zinc, copper, etc. [[6], [7], [8], [9], [10], [11], [12]], phenolic compounds flavonoids [[13], [14], [15]] with antioxidant potentials [[16], [17], [18]]. Amaranth's vitamins are β-carotene (provitamin A), vitamin B6, vitamin C, foliate, and a supplementing source of other vitamins which are thiamine (vitamin B1), riboflavin (vitamin B2), and niacin (vitamin B3), and unsaturated fats such as omega-3 and omega-6. The protein of the leaves of VA is gluten-free, so it will be a promising basis of protein for those who are gluten-sensitive [19].

It contains plentiful antiradical colorant pigments like chlorophyll *a*, β-cyanins, chlorophyll *b*, β-xanthins, and total chlorophyll [[20], [21], [22], [23]] which is unique compared to other leafy vegetables [24]. The colorant pigments could be extracted from VA as environment-friendly natural colorants for food processing industries. It contains numerous antiradical phytochemicals like β-carotene, polyphenols, flavonoid compounds, squalene, tocopherols, and ascorbic acids. Amaranth is regarded as a “superfood” owing to its high nutraceutical values. Amaranth has also been considered a future millennium and climate-resilient food as it is resistant to the changing climate and many abiotic factors. The attractive flavor, taste, color, beneficial bioactive compounds, health-promoting properties, and overall food processing performance of amaranth leaves have been subjected to considerable research interest in this crop.

Amaranth is the most significant vegetable grown in Bangladesh and the Indian subcontinent. Amaranth is the crop of the Amaranthaceae family, with chromosomal numbers 2n = 32, 34, under the genus *Amaranthus* [25]. VA leaves provide protein, carotene, and dietary fiber at a reasonable cost [26]. VA is an herbaceous C_4_ dicotyledonous plant with over 70 species, 17 of which yield edible leaves and 3 of which yield food grains [27]. Although it is thought to have originated in South or Southeast Asia, VA can now be initiated in tropical and temperate climates all over the world [28]. VA is a flexible crop demonstrating high adaptability to diverse habitats, even severe biotic and abiotic factors [[29], [30], [31]].

The intensity of genetic diversity in a crop plant measures its genetic dynamism. Plant breeding is based on selection, which can only be done efficiently if desirable features are present. Consequently, the achievement of breeding is entirely dependent on variety. Exploiting the hereditary variation of available germplasm to select high-yielding genotypes along with high colorant pigments and antiradical phytochemicals that may produce a sufficient yield along with these nutraceuticals is the key tactics used by plant scientists to tackle the difficulty of achieving a higher yield [32].

Variability emerges owing to environmental and genetic factors. Additive and non-additive variability are the two types of variability. The phenotypic variance has been divergent into the environmental and genotypic variance to provide sufficient knowledge of the variations pattern. The intensity of diversity in any feature is critical for crop enhancement through breeding. Genetic diversity determines the achievement of any crop development operation [33]. We hypothesize that our tested genotypes may have a wide array of differences along with high yield, high colorant pigments, and antiradical phytochemical potential. Considering the above facts, this study was performed with the following objectives: to investigate the variations among VA genotypes with respect to FY, related morphological traits, bioactive colorant pigments, and antiradical phytochemicals and to select high colorant pigments and antiradical phytochemical enrich high yielding VA genotypes based on association, variability, and PC of component traits.

## 2 Materials and method

### 2.1 Experimental site

The test site is situated in the center of Madupur Tract, with a latitude of approximately 24° 23′, a longitude of 90° 08′, and an average elevation of 8.4 m above sea level. The soil conditions were slightly acidic (pH 6.4), low in organic matter (0.87%), total N of 0.09%, and exchangeable K of 0.13 cmol kg^−1^.

### 2.2 Materials, design, and layout

Seeds of 20 genotypes of VA were collected from the Genetics and Plant Breeding Department in Bangabandhu Sheikh Mujibur Rahman Agricultural University (BSMRAU) Salna, Gazipur-1706, Bangladesh. The study was executed using 20 promising genotypes of VA (AA1, AA2, AA3, AA4, AA5, AA6, AA7, AA8, AA9, AA10, AA11, AA12, AA13, AA14, AA15, AA16, AA17, AA18, AA19, and AA20). The study was executed at BSMRAU with three replications in a randomized complete block design (RCBD). Three replications were executed by dividing the experimental field into three blocks. Each block was also subdivided into twenty experimental plots/experimental units. The experimental unit was 1 m × 1 m. The row-to-row and plant-to-plant spacings were 25 cm and 5 cm, respectively.

### 2.3 Land preparation and fertilization

The experimental site was located at a lower altitude with well irrigation facilities. The experimental field's ground was plowed with a tractor-drawn disc plow and then harrowed. The land was thoroughly prepared by plowing and cross-plowing three to four times, then laddering to achieve good tilt conditions. Stubbles and weeds were cleared and the area was ultimately prepared by applying a recommended base dose of fertilizers. Recommended chemical fertilizer and compost were applied. In a final land preparation, 10 t/ha compost was applied. Urea, TSP (triple super phosphate), murate of potash (MP), and gypsum were applied @, 200, 100, 150, and 30 kg/ha, respectively.

### 2.4 Intercultural operation and aftercare

During the cropping time, necessary intercultural operations were performed to ensure the plants' healthy growth and development. Weeding and hoeing were done every seven days intervals. Irrigation was scheduled every 5–7 days intervals.

### 2.5 Data collection

After 30 days of seed sowing, data were randomly collected from 10 plants for each trait of all replications. Without any loss of roots, plants were uprooted from the soil. At the base of the stem (base of ground level), the plants were cut into separate shoots.

#### 2.5.1 Morpho-agronomical data

##### 2.5.1.1 Plant height (cm)

It was measured in centimeters (cm) from the base to the peak of the stem.

##### 2.5.1.2 Stem base diameter (mm)

It was measured in millimeters using digital slide calipers at the base of the stem.

##### 2.5.1.3 Leaves per plant (L/P)

Ten plants were randomly selected and all leaves were physically counted. The average number was obtained by dividing the total number by 10.

##### 2.5.1.4 Shoot weight (g)

The shoot weights (stem + leaves) of ten randomly chosen plants were measured in grams and divided by ten to get the average.

##### 2.5.1.5 Root weight (g)

The root weights of ten randomly chosen plants were measured in grams which were then divided by ten to get the average.

##### 2.5.1.6 Stem weight (g)

The stem weights of ten randomly chosen plants were measured in grams which were then divided by ten to get the average.

##### 2.5.1.7 Foliage Yield FY (g)

To calculate FY, leaves of all plants were picked from a 1 m^2^ area and weighted by balance.

#### 2.5.2 Colorant pigments and antiradical phytochemicals

##### 2.5.2.1 Determination of chlorophylls (mg/g)

Chlorophyll *a* (Chl *a*), total chlorophylls, and chlorophyll *b* (Chl *b*) were estimated using a spectrophotometer (Hitachi, Tokyo, Japan) at 663 and 646 nm from acetonic (80%) extracts of fresh amaranth leaves using the Lichtenthaler and Wellburn's method [[34], [35], [36]].

##### 2.5.2.2 Determination of β-cyanins and β-xanthins (ng/g)

β-cyanins and β-xanthins were extracted from fresh amaranth leaves using 80% methanol containing 50 mM ascorbic acid [37,38]. At 540 and 475 nm, β-cyanins and β-xanthins were spectrophotometrically detected. β-Cyanins had a mean molar extinction coefficient of 62 × 106 cm^2^ mol^−1^, while β-xanthins had a mean molar extinction coefficient of 48 × 106 cm^2^ mol^−1^. For β-cyanins and β-xanthins, the data were represented as equivalent to indicaxanthin ng/g fresh weight (FW).

##### 2.5.2.3 Determination of β-carotene (mg/g)

We followed the previously described procedure for extracting and estimating β-carotene [39]. A fresh leaf sample (500 mg) was extracted in 10 mL 80% C_3_H_6_O and to complete the extraction process it was centrifuged for 3–4 min at 10,000 rpm. The supernatant was removed and brought to a volume of 20 mL in a volumetric flask, where the absorbance at 510 and 480 nm was measured with a spectrophotometer (Hitachi, U-1800, Tokyo, Japan).

The β-carotene content was calculated by the following formula:

β-carotene content = 7.6 (Abs. at 480) – 1.49 (Abs. at 510) (1000 fresh weight of leaf taken)/final volume.

##### 2.5.2.4 Determination of TF and TP (GAE μg/g DW)

TF and TP were measured using the aluminum chloride colorimetric technique [40] and the Folin-Ciocalteu reagent [41,42], respectively. In a test tube, leaf extract (500 mL), MeOH (1.5 mL), 10% AlCl_3_6H_2_O (0.1 mL), 0.1 mL CH_3_CO_2_K (1 M), and 2.8 mL distilled water were added to estimate TF. For TP estimation, in a 20 mL test tube, standard solutions for leaf extract (50 L), 1 mL Folin-Ciocalteu reagent (1:4 with deionized water), and 1 mL Na_2_CO_3_ solution (10% wt/vol) were mixed, and allowed to stand at room temperature for 90 min in the dark. A spectrophotometer was utilized to estimate absorbance at 415 and 760 nm. TF and TP were determined using a gallic acid standard curve. The data were expressed as gallic acid equivalent (μ*g*/g DW).

### 2.6 Statistical analysis

Replication-wise means data of a trait was obtained by averaging the sample mean [43,44]. The average data of various traits were analyzed statistically [[45], [46], [47]] and biometrically [48,49]. All mean data obtained for each character were analyzed using Statistix 8 software [[50], [51], [52], [53]]. The mean, range, and standard deviation (SD) for each character were also estimated. Phenotypic variances and error mean sum of squares (MS) were estimated according to Johnson et al. [54]. Phenotypic and genotypic coefficient of variation (GCV and PCV) were analyzed following Burton [55]. The formula followed by Hanson et al. [56] was used to estimate broad-sense heritability (h^2^_b_). The formula followed by Lush [57] was used to estimate the expected genetic advance for traits. The formula followed by Comstock and Robinson [58] was used to estimate the genetic advance in the percentage of the mean for traits. The formula followed by Millar et al. [59] was used to estimate the genotypic and phenotypic correlation coefficient for traits. The formula followed by Wright [60] was used to estimate the PCs for traits. The biochemical and morpho-physiological traits were used to build a two-way clustering heatmap using the R package Complex Heatmap [61] in the R studio software [62]. The principal component analysis (PCA) was used to decrease the dimensionality of the dataset without losing key information. The Eigenvalue, latent vectors and PCA-biplot are extracted from the PCA. The PCA was carried out using the packages ggplot2, factoextra, and FactoMineR [63,64].

## 3 Results and discussion

All examined traits exhibited noteworthy deviations for ANOVA which specified a wide divergence in the traits among accessions. Previous studies also recorded similar variations in VA [[65], [66], [67]], *Zea mays* [[68], [69], [70], [71]], *Oryza sativa* [[72], [73], [74], [75], [76]], *Cocos nucifera* [77,78], *Abelmoschus esculenthus* [79,80], Mungbean [81,82] and broccoli [83] genotypes for yield, its related traits, and bioactive phytochemicals.

### 3.1 Performance of morpho-agronomical traits

The intensity of variation across the genotypes in respect of FY, related morphological traits, bioactive colorant pigments, and antiradical phytochemicals were studied and their grand mean value, standard error, CV, and least significance difference at 1% have been shown in Table 1. The genotypes differed significantly for all traits, indicating wide-ranging variability across the genotypes of the examined traits (Table 1).

**Table 1:** Mean performance and standard deviation of FY, related morphological traits VA.

Genotypes	PH	SBD	L/P	SW	RW	STW	FY
AA1	23.27 ± 1.11	7.51 ± 0.53	9.67 ± 0.44	13.55 ± 0.38	1.02 ± 0.38	5.49 ± 0.33	54.90 ± 0.62
AA2	18.63 ± 0.55	6.09 ± 0.64	8.67 ± 0.29	6.73 ± 0.36	0.43 ± 0.36	2.88 ± 0.17	28.75 ± 0.64
AA3	20.12 ± 0.30	4.98 ± 0.52	9.00 ± 0.40	9.08 ± 0.45	0.87 ± 0.45	4.31 ± 0.26	34.24 ± 0.63
AA4	27.69 ± 1.35	6.50 ± 0.69	9.67 ± 0.49	10.25 ± 0.52	0.62 ± 0.52	4.13 ± 0.25	41.92 ± 0.73
AA5	20.6 ± 1.10	8.92 ± 0.34	10.00 ± 0.44	8.66 ± 0.61	0.62 ± 0.35	3.80 ± 0.23	39.25 ± 1.08
AA6	29.02 ± 0.85	6.99 ± 0.33	9.67 ± 0.49	15.23 ± 0.35	1.16 ± 0.35	7.15 ± 0.43	72.29 ± 1.08
AA7	18.37 ± 0.51	4.25 ± 0.23	7.67 ± 0.40	4.95 ± 0.35	0.48 ± 0.35	2.01 ± 0.12	20.31 ± 0.64
AA8	17.72 ± 0.65	4.60 ± 0.27	8.00 ± 0.43	5.29 ± 0.50	0.39 ± 0.50	1.88 ± 0.11	18.90 ± 0.78
AA9	17.97 ± 0.96	4.55 ± 0.23	9.33 ± 0.40	5.24 ± 0.52	0.38 ± 0.52	2.03 ± 0.12	20.43 ± 0.63
AA10	23.83 ± 1.34	5.99 ± 0.22	9.67 ± 0.37	7.40 ± 0.35	0.61 ± 0.35	3.60 ± 0.22	35.92 ± 0.89
AA11	16.09 ± 0.81	5.67 ± 0.23	8.33 ± 0.40	4.10 ± 0.57	0.18 ± 0.57	1.06 ± 0.06	9.44 ± 0.96
AA12	19.7 ± 0.99	4.36 ± 0.17	10.00 ± 0.40	3.97 ± 0.54	0.37 ± 0.54	1.28 ± 0.08	12.56 ± 0.66
AA13	15.55 ± 1.10	5.77 ± 0.23	8.67 ± 0.38	5.28 ± 0.46	0.49 ± 0.46	3.89 ± 0.23	39.74 ± 0.68
AA14	17.15 ± 0.38	4.30 ± 0.36	6.67 ± 0.38	4.65 ± 0.52	0.35 ± 0.52	1.55 ± 0.09	15.57 ± 0.62
AA15	21.8 ± 1.28	4.52 ± 0.25	8.33 ± 0.40	8.18 ± 0.50	0.57 ± 0.50	3.33 ± 0.20	33.18 ± 0.69
AA16	22.7 ± 1.43	5.76 ± 0.27	8.00 ± 0.38	11.20 ± 0.38	0.76 ± 0.38	4.71 ± 0.28	47.08 ± 0.62
AA17	22.33 ± 0.95	6.60 ± 0.29	11.33 ± 0.40	5.66 ± 0.35	0.37 ± 0.35	2.54 ± 0.15	25.39 ± 0.64
AA18	20.47 ± 0.49	4.55 ± 0.62	9.00 ± 0.39	6.00 ± 0.35	0.40 ± 0.35	3.07 ± 0.18	27.65 ± 0.66
AA19	17.13 ± 0.94	4.39 ± 0.40	6.33 ± 0.40	6.12 ± 0.68	0.20 ± 0.68	3.24 ± 0.19	32.96 ± 0.62
AA20	18.63 ± 0.65	5.40 ± 0.33	8.33 ± 0.36	7.23 ± 0.52	0.53 ± 0.52	2.55 ± 0.15	25.26 ± 0.62
LSD	1.22	0.44	0.43	1.05	0.09	0.55	5.09
SE	0.46	0.16	0.02	0.40	0.03	0.21	1.91
Min	15.55	4.25	6.33	3.97	0.20	1.06	9.44
Max	29.02	8.92	11.33	15.32	1.16	7.15	72.29
GM	20.44	5.59	8.82	7.44	0.54	3.05	31.78
CV (%)	1.68	5.22	5.93	2.97	6.98	1.83	0.21
MSS	**	**	**	**	**	**	**

PH = Plant height, SBD = Stem base diameter, L/P = Leaves/plant, SW = Shoot weight, RW = Root weight, STW = Stem weight, FY = Fresh yield, LSD = Least significant difference, SE = Standard error, CV = Coefficient of variation, ** = Significant at 1% level, MSS = Mean sum of squares, Min = Minimum, Max = Maximum, GM = Grand mean.

#### 3.1.1 Plant height

The substantial differences in genotype were demonstrated in plant height. The plant height of twenty VA genotypes varied from 15.55 cm to 29.02 cm, with a mean performance of 20.44 cm (Table 1). The maximum plant height was recorded in AA6 which was parallel to AA4 thereafter AA10, AA15, AA16, and AA17. Alternatively, plant height was the lowest in AA13. Among 20 genotypes, nine showed mean performance over their mean values. Plant height displayed a 1.68% coefficient of variation (CV). Shukla et al. [84] recorded the greatest plant height of 20.43 cm in AV-17 and the lowermost plant height of 15.41 cm in AV-36 in VA which is in conformity with our present findings.

#### 3.1.2 Stem base diameter (SBD)

SBD differed from 4.25 mm to 8.92 mm along with a mean value of 5.59 mm (Table 1). The maximum SBD was found in AA5 showed which was parallel to AA1 thereafter AA6, AA17, AA4, and AA2. In contrast, genotype AA7 exhibited the lowest SBD. Out of 20 genotypes, ten exhibited above-average mean values. SBD exhibited a CV of 5.22%. Hasan et al*.* [85] found a mean SBD of 11.80 mm–33.20 mm in VA. The differences in SBD may be ascribed because of the differences in varietal genetic makeup, cultivation technique, management process, and deviation of environmental conditions in different locations.

#### 3.1.3 Leaves per plant (L/P)

The mean performance of L/P of 20 VA genotypes ranged from 6.33 to 11.33, with a mean performance of 8.82 (Table 1). Genotype AA17 showed the maximum L/P which was parallel to the genotypes AA5 and A12 thereafter AA1, AA4, AA6, AA10, AA3, and AA18. Alternatively, the minimum L/P was recorded in AA19.14 genotypes out of 20 scored above-average mean performance. The CV of L/P was 5.93%. In VA, Sarker et al*.* [67] reported that L/P ranged from 4.30 to 20.52. Cultivation technique, varietal genetic makeup, management process, and deviation of environmental conditions may influence the differences in L/P compared to our present study.

#### 3.1.4 Shoot weight

Shoot weight varied progressively in different amaranth genotypes which range varied from 3.97 g to 15.32 g with a mean performance of 7.44 g (Table 1). The extremely significant differences in genotype revealed that the genotypes were extremely diverse for shoot weight. The highest shoot weight was obtained from AA6 which was parallel to the genotype AA1. Alternatively, AA12 had the lowest shoot weight. Among 20 genotypes, seven scored above-average mean performance. The CV of shoot weight was 2.97%. However, Shankar et al. [86] observed a higher range of the shoot weight of VA from 11.87 g to 128.7 g may be because of higher harvesting age and deviation in the varietal genetic makeup, management process, and environmental conditions.

#### 3.1.5 Root weight

Genotypes diverged in root weight ranging from 0.20 g to 1.16 g along with an average value of 0.54 g (Table 1). Across the genotypes, the average root weight was the maximum in AA6, which was parallel to the genotypes AA1, while the lowest values were recorded in AA19 (Table 1). Out of 20 genotypes, six showed a mean performance over the average value. The CV of shoot weight was 6.98%.

#### 3.1.6 Stem weight

The highly noteworthy deviation in genotypes was confirmed indicating a huge divergence in stem weight. The stem weight of twenty VA genotypes diverged from 1.06 g to 7.15 g with a mean performance was 3.05 g (Table 1). The genotype AA6 displayed the maximum stem weight which was parallel to the genotypes AA1 thereafter AA16, AA3, and AA4. The lowest stem weight was confirmed in AA5. Out of 20 genotypes, seven genotypes had high values than average mean performance. The CV of stem weight was 1.83%. However, Ahammed et al. [87] noticed a higher range of stem weight from 79.1 to 205.32 g in stem amaranth. It may be because of harvesting at higher ages and deviation in the varietal genetic makeup, management process, and environmental conditions.

### 3.2 Performance of colorant pigments and antiradical phytochemicals

#### 3.2.1 Chlorophyll *a*

Chlorophyll *a* displayed noteworthy differences in genotypes, indicating a huge spectrum of divergence among genotypes. Chlorophyll *a* differed from 0.40 to 0.63 mg/g FW with a mean performance of 0.53 mg/g FW (Table 2). Genotype AA20 displayed the maximum chlorophyll *a* which was parallel to the genotypes AA8, AA10, AA12, AA15, AA16, and AA18. In contrast, genotype AA1 exhibited the lowest chlorophyll *a*. Out of 20 genotypes, 14 scored above-average mean values. Chlorophyll *a* exhibited CV of 0.31%. Khanam and Oba [88] noticed that the chlorophyll concentrations in VA differed from 0.21 to 0 0.44 mg/g FW in VA which was lower than our present study. It may be explained due to deviation in varietal genetic makeup.

**Table 2:** Mean performance and standard deviation of bioactive compounds VA.

Genotypes	Chl *a*	Chl *b*	Total Chl	β-Cy	β-X	β-C	TF	TP
AA1	0.40 ± 0.05	0.16 ± 0.03	0.56 ± 0.08	93.08 ± 1.17	215.51 ± 4.23	0.22 ± 0.02	90.33 ± 3.29	4.51 ± 0.15
AA2	0.52 ± 0.07	0.23 ± 0.04	0.75 ± 0.09	122.40 ± 1.16	266.09 ± 5.28	0.22 ± 0.02	94.32 ± 3.44	15.89 ± 0.40
AA3	0.58 ± 0.03	0.27 ± 0.02	0.85 ± 0.07	184.04 ± 1.19	274.47 ± 5.45	0.21 ± 0.02	89.66 ± 3.26	6.88 ± 0.20
AA4	0.54 ± 0.06	0.23 ± 0.03	0.77 ± 0.07	51.96 ± 1.17	223.10 ± 4.38	0.21 ± 0.02	88.89 ± 3.24	6.22 ± 0.19
AA5	0.54 ± 0.08	0.23 ± 0.03	0.77 ± 0.10	65.06 ± 1.16	269.68 ± 5.35	0.22 ± 0.02	94.55 ± 3.45	4.55 ± 0.16
AA6	0.57 ± 0.07	0.26 ± 0.03	0.83 ± 0.08	63.42 ± 1.20	201.34 ± 3.95	0.22 ± 0.02	89.67 ± 3.27	4.11 ± 0.15
AA7	0.58 ± 0.06	0.27 ± 0.02	0.85 ± 0.08	63.43 ± 1.17	243.51 ± 4.80	0.22 ± 0.02	79.88 ± 2.89	2.90 ± 0.13
AA8	0.59 ± 0.05	0.28 ± 0.03	0.87 ± 0.09	45.14 ± 1.17	198.71 ± 3.89	0.21 ± 0.02	95.67 ± 3.50	62.55 ± 1.47
AA9	0.50 ± 0.05	0.21 ± 0.03	0.71 ± 0.14	95.80 ± 1.21	279.88 ± 5.57	0.21 ± 0.02	86.32 ± 3.14	14.00 ± 0.35
AA10	0.59 ± 0.02	0.28 ± 0.03	0.87 ± 0.09	114.25 ± 1.16	253.29 ± 5.01	0.23 ± 0.02	13.11 ± 1.18	6.22 ± 0.19
AA11	0.53 ± 0.08	0.22 ± 0.03	0.75 ± 0.17	246.61 ± 1.16	312.69 ± 6.28	0.21 ± 0.01	54.39 ± 1.91	49.77 ± 1.17
AA12	0.56 ± 0.08	0.25 ± 0.03	0.81 ± 0.14	58.03 ± 1.16	234.62 ± 4.62	0.21 ± 0.02	75.10 ± 2.71	11.43 ± 0.30
AA13	0.42 ± 0.06	0.15 ± 0.03	0.57 ± 0.10	74.02 ± 1.58	211.55 ± 4.15	0.17 ± 0.01	75.54 ± 2.72	14.74 ± 0.37
AA14	0.50 ± 0.05	0.21 ± 0.03	0.71 ± 0.09	123.70 ± 1.21	237.71 ± 4.68	0.20 ± 0.06	84.43 ± 3.06	17.43 ± 0.43
AA15	0.56 ± 0.08	0.25 ± 0.02	0.81 ± 0.09	46.35 ± 1.16	196.04 ± 3.84	0.19 ± 0.03	94.55 ± 3.45	22.31 ± 0.54
AA16	0.57 ± 0.06	0.26 ± 0.02	0.83 ± 0.14	51.89 ± 1.16	219.49 ± 4.31	0.18 ± 0.02	75.67 ± 2.73	4.11 ± 0.15
AA17	0.42 ± 0.08	0.16 ± 0.03	0.58 ± 0.10	86.03 ± 1.24	258.17 ± 5.11	0.09 ± 0.01	43.67 ± 1.50	1.01 ± 0.10
AA18	0.56 ± 0.06	0.23 ± 0.03	0.79 ± 0.10	133.97 ± 1.17	266.14 ± 5.28	0.21 ± 0.02	45.20 ± 1.56	10.32 ± 0.27
AA19	0.41 ± 0.07	0.14 ± 0.03	0.55 ± 0.11	117.08 ± 1.16	260.34 ± 5.15	0.22 ± 0.02	45.56 ± 1.57	10.77 ± 0.28
AA20	0.63 ± 0.05	0.31 ± 0.03	0.94 ± 0.04	40.31 ± 1.17	198.67 ± 3.89	0.20 ± 0.01	66.33 ± 2.37	19.31 ± 0.47
LSD	0.02	0.02	0.04	17.53	11.02	0.01	7.64	5.26
SE	0.01	0.01	0.02	6.59	4.14	0.00	2.87	1.98
Min	0.40	0.14	0.55	40.31	196.04	0.09	13.11	1.01
Max	0.63	0.31	0.94	246.61	312.69	0.22	95.67	62.55
GM	0.53	0.23	0.76	93.83	241.05	0.20	74.14	14.45
CV (%)	0.31	0.68	0.31	0.10	0.02	0.70	0.02	0.12
MSS	**	**	**	**	**	**	**	**^a^

a Chl = Chlorophyll, β-Cy = β-cyanins, β-X = β-xanthins, β-C = β-carotene, TF = Total flavonoids, TP =Total polyphenols, LSD = Least significant difference, SE = Standard error, CV = Coefficient of variation, ** = Significant at 1% level, MSS = Mean sum of squares, Min = Minimum, Max = Maximum, GM = Grand mean

#### 3.2.2 Chlorophyll *b*

Substantial deviation in genotypes was demonstrated which indicated a huge variation in chlorophyll *b* content. Genotypes diverged in chlorophyll *b* that ranged from 0.14 to 0.31 mg/g with a mean performance of 0.23 mg/g (Table 2). The highest chlorophyll *b* was displayed in AA20 which was parallel to the genotypes AA10 and thereafter AA8, AA3, AA7, AA6, and AA16. Alternatively, the lowest chlorophyll *b* was displayed in AA19. Among 20 genotypes, nine scored above-average mean performance. The CV of chlorophyll *b* was 0.68%. In VA, Khanam and Oba [88] found that the chlorophyll *b* concentrations varied from 0.14 to 0.27 mg/g FW which corroborated our present findings.

#### 3.2.3 Total chlorophyll

Extensive differences in genotypes were reported that indicated a broad array of total chlorophyll divergence among the accessions. The total chlorophyll of twenty VA genotypes differed from 0.55 to 0.94 mg/g, with a mean performance of 0.76 mg/g (Table 2). The highest total chlorophyll was recorded in AA20 which was parallel to the genotypes AA8 thereafter AA10, AA3, AA6, AA7, and AA16. The lowest total chlorophyll was marked in AA19. Among 20 genotypes, 11 exhibited above-average mean performance. The CV of total chlorophyll was 0.31%. Khanam and Oba [88] reported that the total chlorophyll concentrations varied from 0.35 to 0.71 mg/g FW in VA which is lower than the present findings.

#### 3.2.4 β-Cyanins

β-Cyanins varied progressively in 20 amaranth genotypes. The genotypic difference was noteworthy indicating wide variation in β-cyanins. The mean performance of β-cyanins of 20 VA genotypes ranged from 40.31 to 246.61 ng/g with a mean performance of 93.83 ng/g (Table 2). The genotype AA11 exhibited the highest content of β-cyanins which was parallel to the genotype AA3, AA18, AA14, and AA2. Alternatively, the lowest content of β-cyanins was recorded in AA20. Among 20 genotypes, seven displayed mean performance over the average value. The CV of β-cyanins was 0.10%. In VA, Khanam and Oba [88] observed that β-cyanins varied from 182.7 to 784.8 ng/g which is higher than our present findings. The deviations may be because of the variation in the hereditary composition of genotypes. Other factors like management processes, environmental conditions, and geographical locations may contribute to the differences in β-cyanins content.

Chl = Chlorophyll, β-Cy = β-cyanins, β-X = β-xanthins, β-C = β-carotene, TF = Total flavonoids, TP = Total polyphenols, LSD = Least significant difference, SE = Standard error, CV = Coefficient of variation, ** = Significant at 1% level, MSS = Mean sum of squares, Min = Minimum, Max = Maximum, GM = Grand mean.

#### 3.2.5 β-Xanthins

The β-xanthins deviated from 196.04 to 312.69 ng/g with a mean value of 241.05 ng/g (Table 2). Genotype AA11 showed the maximum content of β-xanthins which was parallel to the genotypes AA9 thereafter AA3, AA5, AA18, AA2, and AA19. In contrast, genotype AA15 exhibited the minimum content of β-xanthins. Out of 20 genotypes, seven displayed mean performance over the average value. β-Xanthins exhibited a CV of 0.02%. Khanam and Oba [88] reported that β-xanthins varied from 374.7 to 655.4 ng/g in VA which is greater than the current results. The chromosomal constitution of genotypes may significantly influence the deviations between the two results. Furthermore, factors like management processes, environmental conditions, and geographical locations may contribute to the differences in β-xanthins content.

#### 3.2.6 β-Carotene

Significant differences in genotypes were found indicating a huge divergence in β-carotene variation across genotypes. The mean β-carotene of twenty VA genotypes ranged from 0.09 to 0.23 mg/g with a mean performance of 0.20 mg/g (Table 2). The highest β-carotene was displayed in AA10 which was parallel to AA1, AA2, AA3, AA5, AA6, AA8, and AA19. The lowest β-carotene was recorded in AA17. Among 20 genotypes, 13 displayed mean performance over the average value. The CV of β-carotene was 0.70%. Sarker et al*.,* [67] reported that β-carotene in VA ranged from 0.62 to 1.09 mg/g which was much higher than our present findings. The deviations may arise from the divergence in the hereditary composition of genotypes. Other factors like the management process, environmental conditions, and geographical locations may contribute to the differences in β-carotene content.

#### 3.2.7 Total flavonoids (TF)

Like other traits, total flavonoids also varied expressively in different amaranth genotypes which diverged from 13.11 to 95.67 μg GAE/g DW with a mean of 74.14 μg GAE/g DW (Table 2). A noteworthy deviation in genotypes indicated a wide divergence in TF across accessions. The highest TF was displayed in AA8 which was parallel to AA1, AA2, AA3, AA5, AA6, AA8, and AA15. Alternately, the lowest TF was marked in AA10. Out of 20 genotypes, 14 displayed mean performance over the average value. The CV of TF was 0.02%. Khanam and Oba [88] reported that TF varied from 62.6 to 77.7 μg/g in VA. We found higher TF with great variations in comparison to the findings of Khanam and Oba [88].

#### 3.2.8 Total polyphenols (TP)

ANOVA revealed a noteworthy divergence in the case of total polyphenols. A huge deviation across the accessions for total TP is evidenced by the extremely significant genotypic difference. Genotypes differed in TP that ranged from 1.01 to 62.55 μg GAE/g DW with a mean performance of 14.45 μg GAE/g DW) (Table 2). Across the accessions, AA8 displayed the highest TP, which was parallel to AA11, while the lowest TP was obtained from AA17 (Table 2). Among 20 genotypes, seven revealed mean performance over the average value. The CV of total polyphenols was 0.12%.

#### 3.2.9 Foliage yield (FY) per m^2^ (g)

The noteworthy deviation in genotypes was confirmed indicating a broad spectrum of variation for foliage yield across the accessions. The mean foliage yield of 20 VA genotypes ranged from 9.44 to 72.29 g with a mean performance was 31.78 g (Table 1). The highest foliage yield was recorded in AA6 which was parallel to VA1. Alternatively, the lowest foliage yield was marked in AA11. Among 20 genotypes, seven displayed mean performance over the average value. The CV of FY was 0.21%. Celine et al. [89] reported that the foliage yield per m^2^ ranged from 120 to 1132.5 g which is greater than the current results. The chromosomal constitution of genotypes may significantly influence the deviations between the two results. Furthermore, factors like management processes, environmental conditions, and geographical locations may contribute to the differences in β-xanthins content. Based on foliage yield and related morphological traits, colorant pigments, and antiradical phytochemicals 7 genotypes i.e., AA5, AA6, AA8, AA10, AA11, AA19, and AA20 can be selected for high foliage yield, bioactive colorant pigments and antiradical phytochemicals for use as colorant pigments and antiradical enrich preferable high yielding cultivars. The genotypes containing high colorant pigments may select to extract environment-friendly natural colorants for food processing industries.

### 3.3 Variability and genetic parameters

In a crop improvement program, variability is fundamental for selecting improved desirable genotypes. In crop improvement programs, the selection of superior genotypes entirely depends on the variability of genotypes [[90], [91], [92], [93]]. The extent of genetic erraticism [[94], [95], [96]] and the extent of heritability of desirable traits determine the success of the improvement of crop breeding [97]. For the development of variations to fulfill the current need, variability in the breeding materials is required. Economically significant characteristics are usually quantitative and interact with the growing environment. Consequently, breeders should partition variability into genotypic, phenotypic, and environmental factors when calculating variability. Crop breeders must create variety to succeed. Because quantitative traits like agronomic traits interact with the environment under investigation, it's critical to divide their influences of them into genotypic, phenotypic, and environmental to determine the additive or heritable share of variability. The genotypic and phenotypic variance (Vg, Vp) and (GCV, PCV), h^2^_b_, GA, and GAPM are presented in Table 3.

**Table 3:** Estimation of genetic parameters for FY, related morphological traits, and bioactive compounds in VA.

Characters	$\sigma_{p}^{2}$	$\sigma_{g}^{2}$	PCV	GCV	h^2^_b_	GA	GAPM
Plant height	13.02	12.90	17.65	17.57	99.10	7.37	36.04
Stem base diameter	1.65	1.56	22.97	22.37	94.84	2.51	44.86
Leaves/plant	1.58	1.31	14.26	12.96	82.68	2.14	24.28
Shoot weight	9.7	9.65	41.86	41.76	99.50	6.38	85.81
Root weight	0.07	0.06	47.28	46.76	97.82	0.52	95.27
Stem weight	2.63	2.63	53.13	53.10	99.88	3.34	109.31
Chlorophyll *a*	0.01	0.01	12.71	12.70	99.94	0.14	26.16
Chlorophyll *b*	0.002	0.002	21.10	21.09	99.90	0.10	43.42
Total chlorophyll	0.01	0.01	15.17	15.17	99.96	0.24	31.24
β-cyanins	2693.39	2693.38	55.31	55.312	100.00	106.91	113.94
β-xanthins	1063.50	1063.50	13.53	13.53	100.00	67.18	27.87
β-carotene	0.001	0.001	15.13	15.11	99.79	0.06	31.09
TF	511.40	511.40	30.50	30.50	100.00	46.59	62.83
TP	242.89	242.89	107.84	107.84	100.00	32.11	222.16
FY per m^2^	227.26	227.26	47.44	47.44	99.99	31.05	97.72

$\sigma_{p}^{2}$ = Phenotypic variance, $\sigma_{g}^{2}$ = Genotypic variance, PCV = Phenotypic coefficient of variation, GCV = Genotypic coefficient of variation, h^2^_b_ = heritability in broad sense, GA = Genetic advance, GAPM = Genetic advance in per-cent of mean.

Phenotypic variance (σ^2^_p_) and genotypic variance (σ^2^_g_) recorded for all the traits were very close or similar indicating the absence of environmental influence on the expression of these traits, i.e., additive genes played a significant role in the expression of these traits. So, the selection of these traits could significantly contribute to the quality and foliage yield of amaranth. In the current study, β-cyanins displayed the maximum GCV and PCV there after β-xanthins, total flavonoids, TP, and FY, signifying that the parameters under study had an extensive divergence and had a greater scope of selection for improving amaranth genotypes. Plant height, shoot weight, stem weight, SBD, and L/P showed moderate GCV and PCV, and these parameters may be selected to improve the examined amaranth genotypes. Low GCV and PCV were noted in root weight, chlorophyll *a*, chlorophyll *b*, total chlorophyll, and β-carotene. The remaining traits had parallel GCV and PCV, indicating a superiority of the effects of the additive gene for these traits, i.e., the expression of these features was a minor consequence of environment or most of the phenotypic variance was genetic in nature, facilitating more opportunity for amaranth development via selection. For all of the features tested, PCV was to some extent larger than GCV. This suggests that the environment governed the phenotype to some extent. Among all attributes, GCV and PCV of TP were both high thereafter β-cyanins, stem weight, and FY per m^2^mentioning the existence of a broad divergence for the features studied and had a larger selection window for amaranth genotype development. The lowest PCV and GCV values were noted for chlorophyll *a*, β-xanthins, β-carotene, and total chlorophyll. When it comes to determining the heritable fraction of variation, variability alone isn't very helpful. The intensity of achievement awaited from a selection is measured by the trait's heritability and genetic progress. Heritability is a term that is commonly used to describe the intensity to which a character can be passed from generation to generation. Information about a character's heritability is significant since it suggests the feasibility and extent of improvement via selection [67]. High heritability, alternatively, is insufficient to achieve considerable improvement via selection in advanced generations unless it is assisted by a significant degree of hereditary progress [67].

β-Cyanins, β-xanthins, TP, and showed 100% heritability indicating the absence of environmental influence in the expression of these traits, i.e., these traits are fully additive in nature. We know that qualitative traits are controlled by mono or oligogenes and have very minor or no environmental influence on their expression. For this reason, all the qualitative traits studied in the experiment had high heritability (᷉⁓ 100%). Hence, the selection of these quality traits could noteworthy improve the food values of our daily diet by developing high nutritious amaranth variety. All the traits had high heritability values and were moderate for L/P. The highest GAPM was noted for TP (222.16) thereafter β-cyanins (113.94), stem weight (109.31), FY per m^2^ (97.72), root weight (95.27), and shoot weight (85.81) whereas, the lowest GAPM was noted for L/P (24.28), chlorophyll *a* (26.16) and β-xanthins (27.87). TF All the traits showed high heritability along with high to moderate GAPM. So, all traits may be selected for the economical and practical enhancement of FY of VA. According to Bhargava et al. [98], the yield of VA has the highest heredity. Ahammed et al. [99] found that *Amaranthus* sp. has a high heritability value in terms of yield per hectare. Lohithaswa et al. [100] found a significant diversity in the grain yield of *Amaranthus* spp both at the phenotypic and genotypic levels.

### 3.4 Correlation

Yield is the ultimate product that is influenced by several interconnected quantitative characteristics. Consequently, yield selection may be ineffective unless other yield components that impact it directly or indirectly are included. When selection pressure is applied to improve a character that is linked strongly to yield, it has an impact on several other connected characters. Therefore, understanding traits and yields and their correlationships are a guide for plant breeders to make improvements in selection concerning a fair understanding of the participation of genetic and non-genetic elements in generating the association [101]. The genotypic correlation coefficients (r_g_) were larger than the phenotypic correlation coefficients (r_p_) in the current study, representing that the viewed relationships among the various traits were due to hereditary causes and under the impact of the environment, the phenotypic expression of correlations is reduced. Estimates of r_g_ and r_p_ between traits are put in Table 4, Table 5. The r_g_ and r_p_ between yield and its related characters were estimated and the findings are put in Table 3. FY had significant and positive association with plant height (r_g_ = 0.73** and r_p_ = 0.73**), SBD (r_p_ = 0.59** and r_g_ = 0.61**), L/P (r_p_ = 0.26* and r_g_ = 0.27*), shoot weight (r_p_ = 0.93** and r_g_ = 0.93**), root weight (r_p_ = 0.85** and r_g_ = 0.86**), stem weight (r_p_ = 0.86** and r_g_ = 0.86**). Alternatively, it was negatively correlated with β-cyanins (r_p_ = −0.31* and r_g_ = −0.31*), β-xanthins (r_p_ = −0.43** and r_g_ = −0.43**) and total polyphenols (r_p_ = −0.49** and r_g_ = −0.49**). The correlation coefficient revealed that the genotype selected on plant height, leaves/plant, shoot weight, SBD, root weight, and stem weight was directly associated with the FY of amaranth genotypes.

**Table 4:** Coefficient of genotypic correlation among FY, related morphological traits, and bioactive compounds in VA.

Characters	SBD	L/P	SW	RW	STW	Chl a	Chl b	T-Chl	β-Cy	β-X	β-C	TF	TP	FY
PH	0.50**	0.55**	0.80**	0.73**	0.66**	0.18	0.20	0.19	−0.34**	−0.34**	−0.00	0.04	−0.48**	0.73**
SBD		0.61**	0.60**	0.53**	0.23	−0.19	−0.15	−0.18	−0.11	0.00	−0.09	0.12	−0.32*	0.61**
L/P			0.27*	0.36**	0.13	−0.06	−0.00	−0.04	−0.14	0.08	−0.35**	−0.07	−0.36**	0.26*
SW				0.93**	0.82**	0.04	0.10	0.06	−0.25	−0.41**	0.16	0.30*	−0.41**	0.93**
RW					0.79**	0.15	0.23	0.18	−0.26*	−0.44**	0.17	0.35**	−0.44**	0.86**
STW						−0.10	−0.05	−0.08	−0.15	−0.45**	0.05	0.07	−0.39**	0.86**
Chl a							0.98**	0.99**	−0.14	−0.14	0.40**	0.08	0.21	−0.12
Chl b								0.99**	−0.19	−0.21	0.39**	0.11	0.20	−0.01
T-Chl									−0.16	−0.17	0.40**	0.10	0.21	−0.10
β-Cy										0.79**	0.15	−0.32	0.22	−0.31*
β-X											0.07	−0.34**	−0.01	−0.43**
β-C												0.18	0.16	0.09
TF													0.12	0.18
TP														−0.49**

PH = Plant height, SBD = Stem base diameter, L/P = Leaves/plant, SW = Shoot weight, RW = Root weight, STW = Stem weight, Chl = Chlorophyll, T-Chl = Total chlorophyll, β-Cy = β-cyanins, β-X = β-xanthins, β-C = β-carotene, TF = Total flavonoids, TP = Total polyphenols, FY=FY, r_g_ = genotypic correlation coefficient, ** = 1% level of probability, * = 5% level of probability.

**Table 5:** Coefficient of phenotypic correlation among FY, related morphological traits, and bioactive compounds in VA.

Characters	SBD	L/P	SW	RW	STW	Chl a	Chl b	T-Chl	β-Cy	β-X	β-C	TF	TP	FY
PH	0.49**	0.50**	0.80**	0.71**	0.66**	0.18	0.20	0.19	−0.33**	−0.34**	0.00	0.04	−0.48**	0.73**
SBD		0.54**	0.58**	0.50**	0.23	−0.19	−0.15	−0.17	−0.11	0.00	−0.08	0.12	−0.31*	0.59**
L/P			0.26*	0.32*	0.12	−0.06	−0.00	−0.03	−0.13	0.07	−0.32*	−0.07	−0.33*	0.24
SW				0.92**	0.82**	0.04	0.10	0.06	−0.25	−0.41**	0.16	0.30*	−0.41**	0.93**
RW					0.78**	0.15	0.23	0.18	−0.26*	−0.43**	0.17	0.35**	−0.44**	0.85**
STW						−0.10	−0.05	−0.08	−0.15	−0.45**	0.05	0.07	−0.39**	0.86**
Chl a							0.98**	0.99**	−0.14	−0.14	0.40**	0.08	0.21	−0.12
Chl b								0.99**	−0.19	−0.21	0.39**	0.11	0.22	−0.08
T-Chl									−0.16	−0.17	0.40**	0.10	0.21	−0.10
β-Cy										0.79**	0.15	−0.32*	0.22	−0.31*
β-X											0.07	−0.34**	−0.01	−0.43**
β-C												0.18	0.16	0.09
TF													0.12	0.18
TP														−0.49**

PH = Plant height, SBD = Stem base diameter, L/P = Leaves/plant, SW = Shoot weight, RW = Root weight, STW = Stem weight, Chl = Chlorophyll, T-Chl = Total chlorophyll, β-Cy = β-cyanins, β-X = β-xanthins, β-C = β-carotene, TF = Total flavonoids, TP = Total polyphenols, FY=FY, r_p_ = phenotypic correlation coefficient, ** = 1% level of probability, * = 5% level of probability.

### 3.5 Path coefficient (PC) analysis

Path coefficient analysis is used in any plant breeding program to persuade the mood of the correlations between yield and its contributing traits that might be utilized as a selection criterion to improve crop production [102]. The purpose of path analysis is to explain the correlation of traits based on a model and effect relationships for assessing the value of the influenced qualities on a given character [103]. When there are more variables in a correlation study, the indirect relationship gets more complicated and essential. In this case, Path coefficient analysis determines the direct and indirect sources of relationships. At the genotypic level, path coefficient analysis allows for a careful assessment of specific components that work to produce a given association and a measurement of each factor's relative relevance [104]. FY per m^2^ was treated as a resultant (dependent) variable whereas, plant height, leaves/plant, shoot weight, SBD, root weight, stem weight, chlorophyll *a*, chlorophyll *b*, total chlorophyll, β-cyanins, β-xanthins, β-carotene, TF, and TP were causal (independent) variables. Assessment of the direct and indirect effect of PC analysis for VA is put in Table 6. PC analysis discovered that root weight (0.51) showed a maximum + ve direct effect on FY thereafter stem weight (0.50), SBD (0.29), and shoot weight (0.11). It implies that these are the leading contributors to FY and that they might be utilized as screening standards for improved FY. Again, the highest negative direct effect on stem yield per m^2^ was obtained by β-cyanins (−0.39) and thereafter plant height (−0.09). The highest indirect positive effect on FY was confirmed in total chlorophyll (18.09) thereafter chlorophyll *b* (17.79) and chlorophyll *a* (11.05) which could be in consideration for the prospective trial program. In any plant breeding, it is very difficult to fully understand the characteristics of all yield components. The residual effect allows an accurate explanation of the pattern of interaction of other possible components of yield that were not included in the investigation of the dependent variables. The residual effect was 0.01084, prevailing the contribution of component characters on FY per m^2^ was 98.92% by the 14 characters studied in path analysis, the rest of 1.08% was the contribution of other factors that were not included in the dependent variable investigation**.**

**Table 6:** Partitioning of r_g_ into direct (bold phase) and indirect effects of FY, related morphological traits, and bioactive compounds in VA.

Characters	PH	SBD	L/P	SW	RW	STW	Chl a	Chl b	T-Chl	β-Cy	β-X	β-C	TF	TP	GCFY
PH	**−0.09**	0.14	−0.05	0.09	0.37	0.33	3.18	2.26	−5.51	0.13	−0.07	−0.00	−0.01	−0.06	0.73**
SBD	−0.04	**0.29**	−0.05	0.07	0.27	0.12	−3.44	−1.74	5.16	0.04	0.00	−0.01	−0.02	−0.04	0.61**
L/P	−0.05	0.18	**−0.09**	0.03	0.18	0.06	−1.09	−0.03	1.05	0.05	0.02	−0.02	0.01	−0.04	0.26*
SW	−0.07	0.17	−0.02	**0.11**	0.47	0.41	0.74	1.07	−1.90	0.10	−0.08	0.01	−0.04	−0.05	0.93**
RW	−0.06	0.15	−0.03	0.10	**0.51**	0.39	2.68	2.60	−5.41	0.10	−0.09	0.01	−0.05	−0.05	0.86**
STW	−0.06	0.07	−0.01	0.09	0.40	**0.50**	−1.79	−0.58	2.33	0.06	−0.09	0.00	−0.01	−0.05	0.86**
Chl a	−0.02	−0.06	0.01	0.01	0.08	−0.05	**18.15**	11.05	−29.35	0.05	−0.03	0.03	−0.01	0.03	−0.12ns
Chl b	−0.02	−0.04	0.00	0.01	0.12	−0.03	17.79	**11.28**	−29.26	0.07	−0.04	0.03	−0.02	0.02	−0.01ns
T-Chl	−0.02	−0.05	0.00	0.01	0.09	−0.04	18.09	11.20	**−29.46**	0.06	−0.03	0.03	−0.01	0.03	−0.10ns
β-Cy	0.03	−0.03	0.01	−0.03	−0.13	−0.07	−2.46	−2.09	4.62	**−0.39**	0.15	0.01	0.05	0.03	−0.31*
β-X	0.03	0.00	−0.01	−0.04	−0.22	−0.22	−2.59	−2.38	5.07	−0.31	**0.190**	0.01	0.05	−0.001	−0.43**
β-C	0.00	−0.03	0.03	0.018	0.09	0.02	7.26	4.41	−11.73	−0.06	0.01	**0.07**	−0.03	0.02	0.09ns
TF	−0.00	0.04	0.01	0.03	0.18	0.04	1.53	1.29	−2.87	0.12	−0.07	0.01	−**0.14**	0.02	0.19ns
TP	0.04	−0.09	0.03	−0.05	−0.23	−0.19	3.83	2.26	−6.13	−0.09	−0.00	0.01	−0.02	**0.12**	−0.49**

PH = Plant height, SBD = Stem base diameter, L/P = Leaves/plant, SW = Shoot weight, RW = Root weight, STW = Stem weight, Chl = Chlorophyll, T-chl = Total chlorophyll, β-Cy = β-cyanins, β-X = β-xanthins, β-C = β-carotene, TF = Total flavonoids, TP = Total polyphenols, GCFY = genotypic correlation with FY.

### 3.6 Heatmap and hierarchical clustering for morpho-agronomical and biochemical parameters

**Fig. 1:**
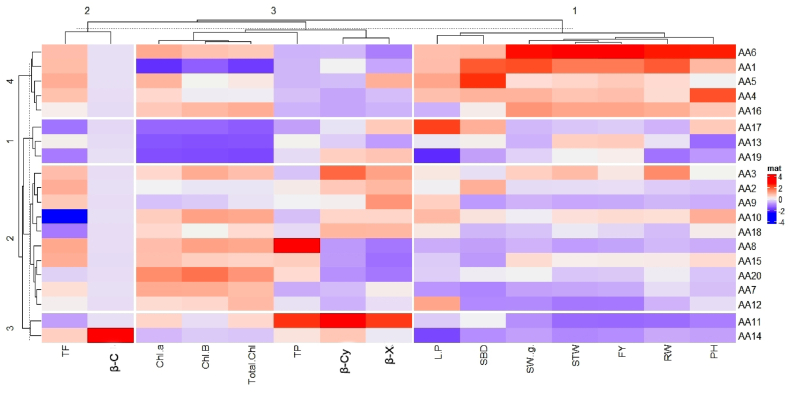
Heatmap and hierarchical clustering for morpho-agronomical and biochemical parameters constructed using Complex Heatmap package. The heatmap plot describes the relative abundance of each amaranth genotype (columns) within each feature (rows). The color code (blue to dark red) displays the row z-score: red color indicates high abundance and blue color low abundance. The dendrogram shows hierarchical clustering of amaranth genotypes based on Euclidian as the measure of distance and Ward's cluster agglomeration method. PH = Plant height, SBD = Stem base diameter, L/P = Leaves/plant, SW = Shoot weight, RW = Root weight, STW = Stem weight, Chl = Chlorophyll, β-Cy = β-cyanins, β-X = β-xanthins, β-C = β-carotene, TF = Total flavonoids, TP = Total polyphenols, FY = Foliage yield.

The heatmap analysis constructed double dendrograms, the dendrogram in the horizontal direction, an arrangement that represents the amaranth accessions, and the 2nd dendrogram in the vertical direction representing traits (Fig. 1). The accessions clustered according to 15 traits demonstrated the trait variability for each accession (Fig. 3). Hierarchical cluster analysis (HCA) divided the traits into three distinct groups, Cluster 4 included traits representing the relative response to agronomical traits (PH, RW, FY, STW, SW, SBD, and L/P). Cluster 3 included biochemical traits such as β-Cy, β-X, β-C, and TP and also the lowest for morpho-agronomical traits. The maximum chlorophyll found in cluster 2. A heatmap is a data imagining practice that displays the extent of a phenomenon as color in two dimensions. The color variation may be by hue or intensity, giving noticeable visual indications to the reader about how the phenomenon is clustered or varies over space. It visualizes the relative patterns of high-abundance features against a background of features that are mostly low abundance or absent. A combined heatmap with HCA is a data visualization method that can be useful in the exploration of complex relationships between multiple parameters under multiple conditions.

### 3.7 Principal component analysis (PCA)

**Fig. 2:**
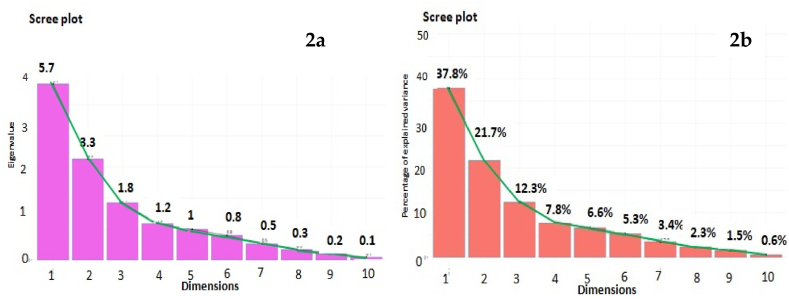
Scree plot constructed for 15 morpho-agronomical and biochemical. (a) Eigenvalues of top ten PCs (b) Percentage of variance of ten PCs.

**Fig. 3:**
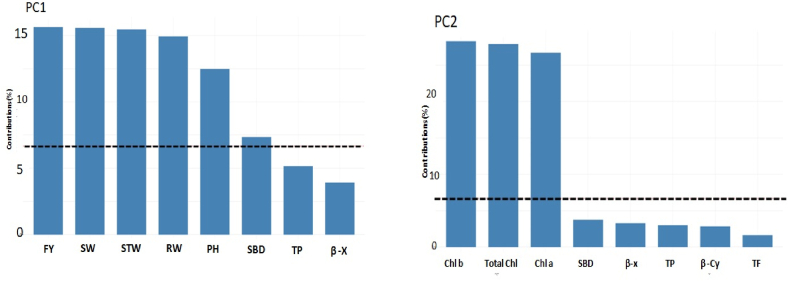
Bar graph showing the contribution of variables to PC1 and PC2. PH = Plant height, SBD = Stem base diameter, SW = Shoot weight, RW = Root weight, STW = Stem weight, FY = foliage yield, Chl = chlorophyll, β-Cy = Chl *a* = Chlorophyll *a*, Chl *b* = Chlorophyll *b*, β-Cy = β-cyanins, β-X = β-xanthins, TF = Total flavonoids, TP = Total polyphenols.

**Fig. 4:**
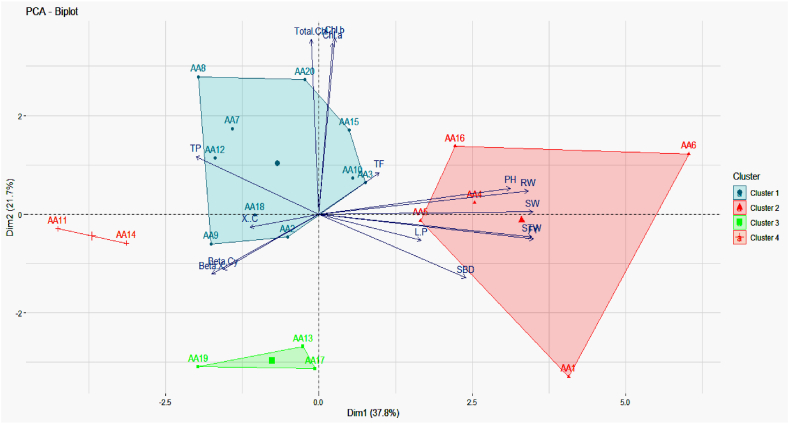
PCA-Biplot of amaranth genotypes. Genotypes are dispersed in different ordinates based on their dissimilarity among them. The length and color intensity of a vector in the biplot indicates the quality of representation and the contribution of the traits, respectively, to the principal components. The angles between the vectors derived from the middle point of biplots exhibit positive or negative interactions of studied traits. Bigger shapes indicate the centroid of the corresponding cluster. PH = Plant height, SBD = Stem base diameter, L/P = Leaves/plant, SW = Shoot weight, RW = Root weight, STW = Stem weight, Chl = Chlorophyll, β-Cy = β-cyanins, β-X = β-xanthins, β-C = β-carotene, TF = Total flavonoids, TP = Total polyphenols and FY = Foliage yield.

The percentage of variation explained by the five three principal components (PC) and the vector loadings for each agronomic character and PC were eternized (Fig. 2, Fig. 3). The Eigenvalues for PC1, PC2 PC3, PC4, and PC5 were 5.7, 3.3, 1.8, 1.2, and 1 respectively (Fig. 2a). These five components alone furnished a maximum of 86.2% of the variability among the genotypes (Fig. 2b). The first principal component PC 1 interpreted for 37.8% of the total variation and the PC1 contribution on five traits *viz*., FY, SW, STW, RW, and PH (Fig. 3). The second principal component PC 2 accounted for 21.7% of the total variation mainly attributed by Chl *b*, total chl, and Chl *a*. PCA is beneficial for the breeders to conduct specific breeding programs according to good information about the groups where certain traits are more important. The degree and path of the contribution of diverse traits in the dissimilar principal components are shown in Fig. 4. Based on PCA for biplot, factor 1 was more contributed by PH, RW, SW, STW, FY, SBD, and LP, while factor 2 is contributed through total chl, Chl *a*, Chl *b,* and TF as shown in Fig. 4. Furthermore, a scatter plot based on the first two factors presented in Fig. 4 showed that the genotypes *viz*., AA20, AA8, and AA15 were present in the extreme upper right side of the scattered plot due to response towards Total chl, Chl *a*, Chl *b,* and TF. Similarly, the genotypes AA6, AA16, and AA5 on the lower right side of the scattered plot were more contributed through PH, RW, SW, STW, FY, SBD, and LP.

## 4 Conclusions

The ANOVA of all attributes examined was significant and showed wide deviations among the 20 genotypes of the VA. All accessions had adequate bioactive compounds, such as antiradical phytochemicals like TP, total flavonoid compounds, β-carotene; and colorant pigments like β-xanthins, chlorophyll *a*, β-cyanins, total chlorophyll, chlorophyll *b*, with extensive variations across genotypes. VA is the only basis of colorant pigments. Taken together of all genetic parameters, genotypes can be chosen on β-cyanins, plant height, β-xanthins, leaves per plant, TF, shoot weight, SBD, root weight, TP, stem weight, chlorophyll *a*, β-carotene, chlorophyll *b*, total chlorophyll, which ultimately suggestively advance the FY of amaranth. However, a correlation study exposed that the progress of FY of VA can be accomplished by selecting root weight, shoot weight, stem weight, plant height, and stem base diameter. Taken together, high direct effects and significant correlation indicated that direct selection through root weight, SBD, stem weight and shoot weight would significantly raise the FY of amaranth genotypes. Seven genotypes (AA5, AA6, AA8, AA10, AA11, AA19, and AA20) may be selected for high FY, bioactive colorant pigments, and antiradical phytochemicals for use as colorant pigments and antiradical enrich preferably high yielding cultivars. Moreover, PCA gave optimum information for the evaluation of genetic diversity among the genotypes and also aids in identifying the genotypes to be further analyzed at the genetic level. The genotypes containing high colorant pigments may select to extract environment-friendly natural colorants for food processing industries. We also can recommend genotypes with moderate FYs and antiradical pigments and phytochemicals for parent materials in future breeding programs.

## Author contribution statement

Conceived and designed the experiments and Performed the experiments: Nishat Jahan and Umakanta Sarker.

Analyzed and interpreted the data: Umakanta Sarker, Nishat Jahan, Mohammad Golam Azam, Daoud Ali, Sezai Ercisli, Mohammad Mehfuz Hasan Saikat, Md. Motaher Hossain and Kirill S. Golokhvast.

Contributed reagents, materials, analysis tools or data: Umakanta Sarker, Nishat Jahan and Mohammad Golam Azam.

Wrote the paper: Nishat Jahan, Umakanta Sarker, Mohammad Golam Azam, Daoud Ali, Sezai Ercisli, Mohammad Mehfuz Hasan Saikat, Md. Motaher Hossain and Kirill S. Golokhvast.

## Data availability statement

Data included in article/supplementary material/referenced in article.

## Additional information

No additional information is available for this paper.

## Declaration of competing interest

The authors declare that they have no known competing financial interests or personal relationships that could have appeared to influence the work reported in this paper.
